# Chromatin Configuration in Diplotene Mouse and Human Oocytes during the Period of Transcriptional Activity Extinction

**DOI:** 10.3390/ijms241411517

**Published:** 2023-07-15

**Authors:** Irina Bogolyubova, Daniil Salimov, Dmitry Bogolyubov

**Affiliations:** 1 Institute of Cytology of the Russian Academy of Sciences, 194064 St. Petersburg, Russia; ibogol@mail.ru; 2 Clinical Institute of Reproductive Medicine, 620014 Yekaterinburg, Russia; dfsalimov@mail.ru

**Keywords:** mammalian oocytes, human oocytes, oocyte nucleus, germinal vesicle, chromatin configuration, transcriptional activity, nucleolus-like body, atypical nucleoli, karyosphere, oocyte quality

## Abstract

In the oocyte nucleus, called the germinal vesicle (GV) at the prolonged diplotene stage of the meiotic prophase, chromatin undergoes a global rearrangement, which is often accompanied by the cessation of its transcriptional activity. In many mammals, including mice and humans, chromatin condenses around a special nuclear organelle called the atypical nucleolus or formerly nucleolus-like body. Chromatin configuration is an important indicator of the quality of GV oocytes and largely predicts their ability to resume meiosis and successful embryonic development. In mice, GV oocytes are traditionally divided into the NSN (non-surrounded nucleolus) and SN (surrounded nucleolus) based on the specific chromatin configuration. The NSN–SN transition is a key event in mouse oogenesis and the main prerequisite for the normal development of the embryo. As for humans, there is no single nomenclature for the chromatin configuration at the GV stage. This often leads to discrepancies and misunderstandings, the overcoming of which should expand the scope of the application of mouse oocytes as a model for developing new methods for assessing and improving the quality of human oocytes. As a first approximation and with a certain proviso, the mouse NSN/SN classification can be used for the primary characterization of human GV oocytes. The task of this review is to analyze and discuss the existing classifications of chromatin configuration in mouse and human GV oocytes with an emphasis on transcriptional activity extinction at the end of oocyte growth.

## 1. Introduction

The problem of female infertility is becoming increasingly widespread in the world, including in connection with the socio-demographic trend of increasing the age of childbearing [1]. In the ART practice, “mature” oocytes, i.e., being at the stage of metaphase II (MII), are generally used, which are determined by the presence of a metaphase plate in the oocyte and the first polar body (PB) in the perivitelline space [2]. The human or any animal oocyte—whether vertebrate or invertebrate—is a highly specialized and unusual cell compared to a somatic cell. It is a giant cell that stores cytoplasmic and nuclear macromolecules for use in the early stages of embryogenesis. The main point is that the growing oocytes—which will be discussed in this review—are in the prophase of meiosis, namely, at the long diplotene stage. For almost 200 years, the nucleus of diplotene oocytes has been known as the germinal vesicle (GV) [3]. In ART clinics, human oocytes with an intact GV are usually disposed of or used for scientific purposes with the informed consent of the patient. However, if the number of mature oocytes obtained as a result of the patient’s hormonal stimulation turns out to be very small, then it becomes necessary to “rescue” GV-oocytes by their in vitro maturation (IVM). This additional ART is called rescue IVM, immature rescue, or GV rescue. GV rescue often leads to good results comparable to the development of oocytes in vivo, in terms of the number of pre-implantation embryos obtained [4]. Currently, IVM is one of the most promising approaches in ART aimed at overcoming the problem of female infertility [5].

In ART clinics, the quality of GV oocytes is assessed at the light optical level using simple non-invasive methods for determining the diameter of the oocyte, the position of the GV (central or peripheral), the morphological parameters of the PB, the granularity of the ooplasm, and so on [6,7]. However, such a morphological assessment is informative rather than prognostic [8].

There is increasing evidence that mammalian oocytes—mouse [9,10,11] and human [12]—possess different competencies for further development depending on the GV structure, which is primarily determined by the specific configuration of the chromatin compartment [13]. At the same time, in contrast to the nomenclature proposed for the mouse, there is no unified nomenclature to describe the chromatin configuration of human GV oocytes. This situation often leads to discrepancies and misunderstandings, the overcoming of which, of course, should expand the scope of the application of mouse oocytes as a model for developing new methods for assessing and improving the quality of human oocytes used in ART. In this review, we analyze the existing classifications of chromatin configuration in diplotene mouse and human oocytes during the period of extinction of transcriptional activity and discuss this problem in a comparative aspect.

## 2. Transcriptional Activity and Chromatin Rearrangement in Mammalian Late Diplotene Oocytes

### 2.1. Chromosome Aggregation and Karyosphere Formation

Morphological transformations of the GV have been described, with varying degrees of detail, for many mammals, including rodents (mouse, vole, rat, golden hamster, and forest dormouse), primates (rhesus monkey, humans), pigs, horses, cattle (cows, buffalo), goat, sheep, rabbit, dog, cat, mink, and ferret; see reviews [13,14,15,16,17] and references therein. In mammalian females, meiosis starts during embryonic development, when the oocytes undergo the leptotene, zygotene, and pachytene stages. Before birth, the oocyte arrests at the diplotene, also called the dictyate stage, for a diffuse state of chromatin [18]. The main tendency of transformations of the chromosomal apparatus in the diplotene oocytes of most mammals is its progressive compaction with the formation of heterochromatic conglomerates that vary in morphology [13].

The chromatin of diplotene mammalian oocytes has a specific structure that correlates with a peculiar pattern of epigenetic modifications—such as histone acetylation, phosphorylation, methylation, glycosylation, ubiquitination, and SUMOylation—which largely determine the quality of oocyte maturation [19]. Furthermore, the low-input genome-wide chromatin conformation capture technology allows for demonstrating the unique organization of chromatin in late mouse oocytes, regulated by Polycomb group proteins (PcG), which is reflected in the formation of specific Polycomb-associated domains (PADs) in the absence of the canonical topologically associated domains (TADs) [20]. Thus, in mammalian (mouse) oocytes, there seem to be profound differences in chromatin structure compared to other vertebrates [21].

In addition to the chromosomes themselves, the nucleoli also undergo a significant reorganization during the diplotene stage of female meiosis in mammals, transforming into homogeneous fibrillar derivatives containing some nucleolar proteins [22,23], but not containing ribosomal RNAs [24]. Experiments with labeled precursors of RNA synthesis clearly showed that they are transcriptionally inactive [25,26].

For decades, these structures in mammalian oocytes have traditionally been referred to as nucleolus-like bodies (NLBs) [27]. These NLBs are now also called atypical nucleoli (ANu) [28]. This category also includes morphologically similar structures—the so-called nucleolus precursor bodies (NPBs)—which are provisional nuclear organelles present in the nuclei of zygotes and early embryos that represent a structural platform for chromatin remodeling [29,30].

Among the studied mammals, the goat is the only animal in which the nucleoli are completely resorbed in fully grown oocytes. In the goat, the chromatin remains in a slightly condensed state even in oocytes ready for ovulation, which significantly distinguishes the goat GV from GVs of other mammals [31]. In most other mammals, nucleolar remnants persist for a long time—until the GV breakdown (GVBD) before the first meiotic (reduction) division—and become incorporated into masses of condensed heterochromatin [13]. At this point, the dynamics of nucleoli in mammalian oogenesis is species-specific [16].

In the GV of the mouse and human—as well as in the pig GV [32,33]—the ANu is very large and the most prominent nuclear organelle with a perfectly regular spherical shape. In mouse and human oocytes, the ANu is clearly visible at the light optical level in unfixed and unstained oocytes, which allows for microsurgical manipulations with them [34]. According to immunocytochemical data, the ANu of various mammals and humans may contain not only typical nucleolar proteins, but also some other proteins that are only indirectly related or have nothing to do with nucleolar functions [32,35,36,37].

Despite longstanding research interest, the molecular composition (proteome) of the ANu is unknown and is unlikely to be determined in the near future. Although atypical nucleoli are relatively easy to biopsy using a micromanipulator with proper skill [34], an individual ANu of mice contains approximately 1.6 ng of protein [38], while micrograms of proteins are required for conventional proteomic analysis. Therefore, an incredible number of animals and oocytes will be required for this kind of analysis, not to mention labor costs [28]. It is clear that such work is impossible in principle on human oocytes.

The first electron microscopic study of late GV oocytes isolated from human antral follicles [25] showed that all chromatin in the studied oocytes is associated with the surface of the ANu. Later, these data were confirmed by an independent group of researchers [26], who also showed that some GVs can contain not only a single large ANu, but also a smaller ANu, which, like the large one, is also incorporated into the heterochromatin mass. However, in parallel, it should be noted that in the case of human oocytes, the amount of material is always limited, and the sample is purely individual, especially in electron microscopic studies.

In addition to condensed chromatin, interchromatin granule clusters (IGCs)—also known as nuclear speckles [39,40,41,42]—can also be observed on the ANu surface [26,43]. At the same time, judging by the results of time-lapse imaging of a human oocyte developing within 49 h in vitro [44], IGCs in the GV are very dynamic and do not form a stable complex with ANu, as it may seem when analyzing static images, but are in contact with the ANu surface only for a short time.

The complex of chromosomes associated with each other in a single compact mass located in a limited space of the GV undoubtedly falls under the definition of the karyosphere—a meiosis-specific and evolutionarily conserved structure, species-specific in its morphology [45,46]. However, in relation to mammalian and human oocytes, the concept of the karyosphere is rarely used [25,47], although some authors [17,48] draw a direct parallel between the chromatin–ANu complex in mammalian oocytes and the karyosphere of other organisms.

**Figure 1:**
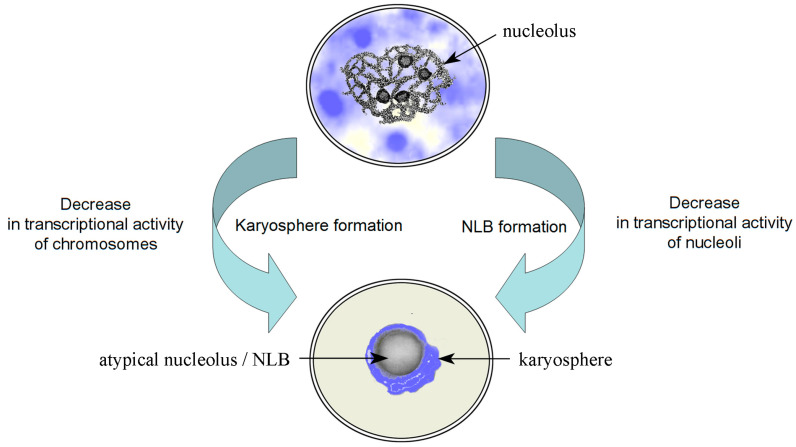
A diagram illustrating the main mechanisms of structural/functional rearrangement of chromatin in the mammalian GV; NLB, nucleolus-like body, aka atypical nucleolus.

Thus, the final stages of oocyte growth in the vast majority of mammals and humans are characterized by specific chromatin condensation and chromosome aggregation around a transcriptionally inert nuclear organelle called the ANu or, formerly, NLB (Figure 1).

### 2.2. Transcriptional Activity of GV Oocytes during Karyosphere Formation

Reducing the intensity of transcriptional activity is a general principle of karyosphere dynamics not only in humans, but also in other vertebrates and invertebrates, in the GV of which the karyosphere is formed [46].

In mouse oocytes, transcriptional activity of the GV gradually decreases as the oocyte grows and chromatin condenses, and this reduction in transcription is associated with the acquisition of meiotic competence by fully grown oocytes [49]. RNAP I- and RNAP II-dependent transcription usually decreases significantly in mouse oocytes with a fully formed karyosphere, while oocytes are transcriptionally active prior to the onset of karyosphere formation [50,51]. As recently shown with ATAC-seq, no new RNA is synthesized in the GV of SN oocytes because RNAP II does not bind to DNA, and the phosphorylation state of RNAP II does not affect its chromatin-binding activity [52]. At the same time, there is a report that Br-UTP—a precursor of RNA synthesis—continues to label newly synthesized transcripts in mouse oocytes with a fully formed karyosphere up to the late stage of antral follicle development [36].

In porcine oocytes, the intensity of RNA synthesis was previously reported to depend on the size of the follicle and the configuration of chromatin in oocytes [53,54,55]. However, recent data indicate that the transcriptional activity of the porcine GV is preserved during oocyte growth [56]. At the same time, in bovine oocytes, RNA synthesis activity decreases during oocyte growth [57,58] and stops in the GV with a well-formed karyosphere [59].

In humans, the smallest GV oocytes are transcriptionally active, as shown in Br-UTP labeling experiments. The level of transcriptional activity decreases with the growth of oocytes and completely stops in large oocytes with a fully formed karyosphere [26]. Similar results were obtained using [3H]-uridine ultrastructural autoradiography, which has shown that the fully formed human karyosphere is completely transcriptionally inert [25]. At the same time, a noticeable incorporation of [3H]-uridine into the karyosphere of human SN oocytes was registered at the light microscopy level [60]. These discrepancies are possibly associated with the presence of different transitional morphological forms of chromatin compaction at the final stages of the development of the karyosphere.

It is assumed that one of the leading factors determining transcriptional repression in fully grown mammalian oocytes is poly(rC)-binding protein 1 (PCBP1). Microinjection of *Pcbp1*-specific siRNAs into fully grown mouse GV oocytes resulted in obvious transcriptional reactivation and a shift in the ratio of chromatin configurations towards the NSN [61]. In addition, embryonic poly(A)-binding protein (EPAB) is also involved in the processes of chromatin reorganization and regulation of transcriptional activity in GV oocytes [62]. The authors showed that transcriptional silencing did not occur in *Epab^–/–^* oocytes, and the reorganization of the SN chromatin configuration and oocyte maturation to MII was rescued by the microinjection of EPAB mRNA into *Epab^−/−^* oocytes.

However, the formation of the karyosphere does not appear to be a prerequisite for the complete suppression of transcription in GV oocytes. For example, in goat oocytes, where the karyosphere does not form, a decrease in GV transcriptional activity is nevertheless recorded as the follicle grows [63], ceasing in oocytes retrieved form the 3 mm follicles [31]. On the contrary, in rabbits, whose oocytes even in primordial follicles have a formed karyosphere, a detectable level of RNA synthesis is maintained for a long period [64].

Experimental data have also confirmed that chromatin condensation and karyosphere formation are not significant transcription suppression factors during mammalian oocyte growth. For example, in preovulatory oocytes obtained from mice with a knockout of *Npm2*—a gene encoding nucleoplasmin 2 that performs various functions as a nuclear chaperone [65]—no karyosphere is formed; nevertheless, transcriptional activity is repressed [66]. Similarly, the experimental decondensation of chromatin in preovulatory mouse oocytes using trichostatin—a deacetylase inhibitor—does not lead to transcriptional reactivation [47].

In summary, the structural transformations of chromatin leading to the formation of the karyosphere generally correlate with the gradual extinction of GV transcriptional activity. Some contradictions in the literature on this issue may be associated with the heterogeneity of the studied oocytes, including their competence for development, which requires verification using modern approaches. Further studies in this direction should also shed light on the question of the presence or absence of obvious causal relationships between the degree of chromatin condensation (karyosphere formation) and the transcriptional activity of the GV.

## 3. Morphodynamics of Heterochromatin in Mouse and Human GV Oocytes

### 3.1. NSN and SN Chromatin Configurations in the Mouse GV

**Figure 2:**
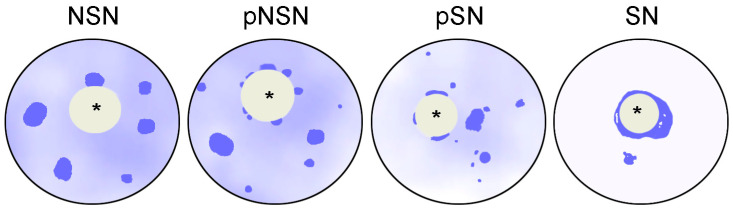
A diagram illustrating the morphological configurations of chromatin in mouse oocytes according to classical/common nomenclature [50,67]. Heterochromatin is shown dark blue; asterisks indicate atypical nucleoli/nucleolus-like bodies.

In the GV of mice, two main types of chromatin configuration are traditionally distinguished according to the degree of its compaction and association with the ANu: NSN (non-surrounded “nucleolus”) and SN (surrounded “nucleolus”) [67]. The heterochromatic “ring” surrounding the ANu in SN oocytes can be most clearly observed on sections (optical or physical) stained with fluorescent, DNA-specific dyes, such as DAPI or Hoechst. Sometimes two intermediate states are distinguished: pNSN (partly NSN) and pSN (partly SN), in which heterochromatin only partially surrounds the ANu [50] (Figure 2 and Table 1). At the same time, some authors [24] do not see the advisability of the special allocation of pNSN configuration, and this, in our opinion, is reasonable. It should be noted that the division of chromatin configurations into NSN–pSN–SN is rather arbitrary due to the dynamics of nuclear structures, and none of these configurations is a discretely stable state, as one might think when analyzing cytological preparations. Nevertheless, the trend of successive transition from NSN to SN configuration can be traced quite clearly during the growth of oocytes and follicles. For example, in the mouse, the SN chromatin configuration begins to be detected only in oocytes larger than 40 µm [68].

As mouse oocytes develop, the number of chromocenters—discrete chromatin structures enriched in tandem DNA repeats and mobile elements of the genome [69]—decreases in the GV. During the NSN–SN transition, the chromocenters are drawn to the ANu periphery, where the centromeric DNA sequences, which occupied the central position in the GV of NSN oocytes, are also located [70]. The ANu-associated chromatin of SN oocytes also contains pericentromeric DNA regions enriched in the major satellite (MaSat), together with which the heterochromatin protein HP1β is concentrated [71].

During the NSN–SN transition in mouse oogenesis, there are close functional relationships between the chromatin-remodeling ATP-dependent helicase ATRX and the H3.3-specific histone chaperone DAXX [72]. ATRX recruits DAXX to the pericentromeric regions during the NSN–SN transition, resulting in both proteins being predominantly concentrated in the ANu-associated heterochromatin compartment of SN oocytes [73,74]. The lack of ATRX leads, in particular, to centromere instability and aneuploidy in mouse embryos [75].

It is important to note that the NSN–SN transition is the main prerequisite for the normal development of the embryo, at least in mice [9,49,76]. After fertilization, only the embryos descending from SN oocytes can develop normally, while the descendants of NSN oocytes stop at the two-cell stage [10,11]. A comparison of the transcriptome profiles of mouse NSN and SN oocytes showed that SN oocytes have increased mRNA levels of many genes required for embryonic cleavage and development [77].

### 3.2. Chromatin Configurations in the Human GV

When doing research related to human ARTs, each group of authors, dealing with a set of GV oocytes obtained from specific patients, often uses their own nomenclature/classification of GV oocytes, without trying to unify it.

**Figure 3:**
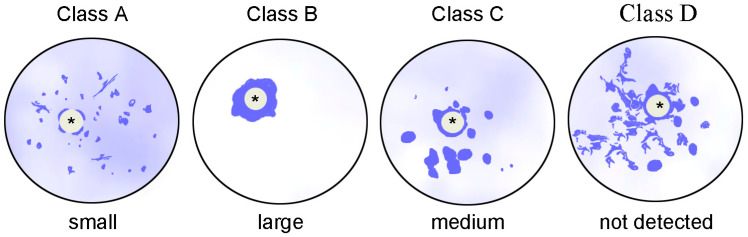
A diagram demonstrating the variety of chromatin configurations in human oocytes at the germinal vesicle (GV) stage The oocyte class is indicated according to the nomenclature proposed by Combelles et al. [78] (up) and Miyara et al. [26] (bottom). Asterisks indicate atypical nucleoli/nucleolus-like bodies.

With regard to human SH oocytes, the classification, which is now the most common, was proposed 20 years ago [78]. In a similar study performed at the same time [25], no special nomenclature was proposed for human GV oocytes, but all oocytes were divided into three size categories: small (104.8 ± 0.96 μm), medium (112.1 ± 2.8 μm), and large (117.7 ± 1.86 μm) (Figure 3 and Table 2).

In both studies, the authors found oocytes with the NSN configuration of chromatin. At the same time, Miyara and co-authors [26] made an important clarification: if all GV chromatin is associated with the ANu (class B oocytes according to Combelles and co-authors [78]), then such oocytes are the largest, while, on the contrary, chromatin is mainly dispersed throughout the GV in the smallest oocytes, even if a Hoechst-positive heterochromatin ring is observed in the GVs.

Apparently, the sequence of classes from A to D presented in the paper [78] does not correspond to the sequence of oocyte growth stages (Figure 3). In addition, if a part of heterochromatin, as a rule, is found to be out of association with the ANu in those mouse SN oocytes in which karyosphere is fully formed, then in human oocytes there is a configuration where all heterochromatin is associated with the ANu [25,26,78].

In the context of determining the quality of human GV oocytes, the relationship between the morphological configuration of chromatin and the success of the oocyte in development is more complex and ambiguous than shown for mouse oocytes, largely due to the lack of criteria for comparing the GVs of laboratory animals (mice) and humans. In addition, researchers working in the field of practical medicine (clinical embryology) often use “new” terms, which can lead to confusion.

For example, the “discovery” in human GV oocytes of a “special” chromosome aggregation phase preceding GVBD (authors’ term gere phase) has been reported [44]. A similar phase of chromosome gathering also exists after the extrusion of the first PB. According to the authors, an “aggregation phase” is not observed in mouse oocytes, and they consider aggregated chromosomes to be a “unique characteristic” of human oocytes [79]. In fact, we are talking about nothing more than the karyosphere, which is formed in both humans and mice, reflecting the common principle of the aggregation of meiotic chromosomes into a single structure at the diplotene stage—in this case around the ANu, called the “central body” of the karyosphere [46].

### 3.3. Is It Possible to Develop a Unified Classification of Chromatin Configurations for Humans and Mice?

As noted above, despite the obvious morphological differences between the configurations of heterochromatin in the GV oocytes of mice and humans, as well as other mammalians, the morphodynamics of the GV, and in particular chromatin, is characterized by general trends briefly described in Section 2.1. In this regard, we believe that a common nomenclature can be developed for heterochromatin configurations in mouse and human oocytes, which can also be extended to other studied mammals.

We propose to take the NSN–SN classification of mouse oocytes, described in Section 3.1, as the basis for such a classification, since the vast majority of mammals and humans, like mice, exhibit an aggregation of oocyte chromosomes around the atypical nucleolus/nucleoli. At the same time, a division of oocytes into traditional four classes—NSN, pNSN, pSN, SN—from our point of view, is difficult to apply in practice and it can confuse the results obtained. This is due to a number of reasons.

First, this classification takes into account only the degree of formation of the heterochromatin rim around the ANu but does not take into account the degree of chromosome aggregation, that is, the completion of the process of karyosphere formation. For example, a pronounced heterochromatin ring can be observed around the ANu in some cases, but at the same time, almost the rest of the chromatin is still diffusely distributed in the nucleoplasm with the preservation of typical chromocenters—that is, the process of karyosphere formation is far from complete.

Second, the foundational studies in this area [50,67,68,80] were carried out using epifluorescence microscopy, while the currently available confocal laser scanning microscopy provides much more informative pictures of the distribution of heterochromatin within the GV. It is often difficult to differentiate between oocytes with typical NSN and pNSN configurations in confocal images, especially at the earliest stages of heterochromatin ring formation.

**Figure 4:**
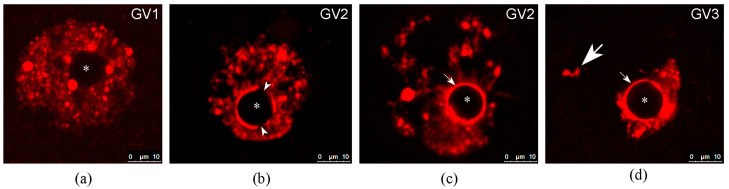
Main chromatin configurations in mouse GV oocytes according to our nomenclature proposed in the present paper. (**a**) GV1—chromatin is diffusely distributed throughout the GV, the heterochromatic rim around the atypical nucleolus is absent. (**b**,**c**) Two variants of GV2—chromatin is still distributed throughout the whole GV and large chromocenters are detected in these cases, as a rule. However, a prominent ring-shaped heterochromatin area is already formed around the atypical nucleolus, being either incomplete (**b**), small arrowheads indicate gaps in the ring) or complete (**c**), small arrow). (**d**) GV3—chromatin is localized in a rather limited area of the GV, corresponding to a formed karyosphere. At the same time, in mice, separate heterochromatic blocks are often also visible outside the karyosphere (large arrow). The small arrow indicates the heterochromatin ring around the atypical nucleolus. Atypical nucleoli are indicated by asterisks in all images. Confocal microscopy, most representative optical sections, a 40× objective; DAPI staining, artificial red color.

In this regard, we propose to subdivide mouse and human GV oocytes into three classes, which correspond to disperse, intermediate, and compact chromatin configurations (Table 3 and Figure 4). In addition, we propose to omit the use of classical names NSN and SN, although the formation of a heterochromatin ring is one of the criteria for the proposed classification (Table 3). First of all, this is due to an understanding of the nature of ANu/NLBs, which do not correspond to the nucleoli either in morphological or in functional characteristics. In addition, the formation of a heterochromatin ring around the ANu cannot be considered as the only criterion for the classification of preovulatory oocytes, as noted above.

Realizing that there is no particular need to escape the widely accepted NSN/SN nomenclature for mice, we nonetheless propose the introduction of a numerical class nomenclature—GV1, GV2, and GV3—which not only better reflects the degree of chromatin condensation and chromosome aggregation, but also facilitates the extension of the proposed classification to other mammalian species. We have to note that for some species, e.g., the pig [54] or monkey [81], this kind of nomenclature is already used for oocytes with different chromatin configurations.

## 4. Conclusions

Despite the variety of morphological patterns of chromatin distribution in human GV oocytes, their assessment, based on the principle of formation of a heterochromatin ring—as in the case of mouse oocytes during the NSN–SN transition, as well as the degree of compaction of chromatin, including karyosphere formation—in our opinion, is quite adequate, simple and convenient both for clinical purposes in ART and in the scientific plan for the search for molecular criteria for oocyte quality.

## Figures and Tables

**Table 1:** Classification of mouse GV oocytes based on morphological chromatin configuration ^1^.

Class	General Characteristics of the GV
NSN	Chromatin is weakly condensed, not associated with atypical nucleoli, and distributed throughout the GV
pNSN	Chromatin is distributed throughout the GV, and only single blocks of condensed chromatin are found in close association with the atypical nucleolus
pSN	Chromatin is distributed throughout the GV, while an incomplete heterochromatin ring or its separate fragments is formed around the atypical nucleolus
SN	The atypical nucleolus is completely surrounded by a heterochromatic ring

^1^ Definitions are presented according to Debey et al. [67] for NSN/SN and Bouniol-Baly et al. [50] for pNSN/pSN.

**Table 2:** Classification of human GV oocytes ^1^.

Class	Characteristics of the GV
According to Combelles et al. [78]:	
A	The atypical nucleolus is only partially surrounded by chromatin; a significant part of the chromatin (“fibrillar chromatin” in the cited paper) is dispersed throughout the GV
B	All chromatin is centered around the atypical nucleolus ^2^
C	The atypical nucleolus is completely surrounded by chromatin, but a significant portion of condensed chromatin (the authors’ “masses of condensed chromatin”) is observed in the rest of the GV
D	The atypical nucleolus is completely surrounded by chromatin, the strands of which (“threads of dispersed chromatin… without any evidence of fibrillar chromatin patterning”) are present in the rest of the GV
According to Miyara et al. [26]:	
Small oocytes	Chromatin is distributed throughout the GV
Oocytes of intermediate size	Condensed chromatin is distributed throughout the GV in the form of large blocks (“aggregates” in the cited paper) and partially surrounds the atypical nucleolus ^3^
Large oocytes	All chromatin is centered around the atypical nucleolus ^4^

^1^ Provided using the authors’ terminology. ^2^ Logically, oocytes of this class should be the latest. ^3^ Judging by the figure presented in the cited paper [26], the heterochromatin ring around the atypical nucleolus is completely formed; GV morphology corresponds to that in class C oocytes according to Combelles et al. [78]. ^4^ GV morphology is identical to that in class B oocytes according to Combelles et al. [78] and also presented in an earlier work [25].

**Table 3:** Proposed summary nomenclature mouse and human GV oocytes, based on morphological chromatin configuration.

Class	Characteristics of the GV			Mouse Traditional Nomenclature [50,67]	Human Nomenclature According to Combelles et al. [78]	Human Nomenclature According to Miyara et al. [26] ^1^
	General	Chromatin State ^1^	Karyosphere State			
GV1	Chromatin is weakly condensed and distributed throughout the GV. Only single blocks of heterochromatin are associated with the atypical nucleolus, or there is no association between chromatin and the atypical nucleolus at all	Disperse chromatin configuration	Absent	NSN + pNSN	A	Small oocytes
GV2	Chromatin is moderately condensed and distributed throughout the GV. An incomplete or complete heterochromatin ring is detected around the atypical nucleolus	Intermediate chromatin configuration	Partially formed	pSN + a portion of SN	C + D	Oocytes of intermediate size
GV3	Heterochromatin is concentrated (completely in humans) in a limited area of the GV around the atypical nucleolus	Condensed/compact chromatin configuration	Fully formed	SN	B	Large oocytes

^1^ The authors did not introduce special names or symbols for the described chromatin configurations.

## Data Availability

The data presented in this study are available on request from the corresponding author.
